# Should capnography be used as a guide for choosing a ventilation strategy in circulatory shock caused by severe hypothermia? Observational case-series study

**DOI:** 10.1186/s13049-017-0357-1

**Published:** 2017-02-15

**Authors:** Tomasz Darocha, Sylweriusz Kosiński, Anna Jarosz, Paweł Podsiadło, Mirosław Ziętkiewicz, Tomasz Sanak, Robert Gałązkowski, Jacek Piątek, Janusz Konstanty-Kalandyk, Rafał Drwiła

**Affiliations:** 1 Severe Accidental Hypothermia Center, Cracow, Poland; 2 Department of Anesthesiology and Intensive Care, John Paul II Hospital, Jagiellonian University Medical College, Cracow, Poland; 3 Polish Medical Air Rescue, Warsaw, Poland; 4 Department of Anesthesiology and Intensive Care, Pulmonary Hospital, Zakopane, Poland Tatra Mountain Rescue Service, Zakopane, Poland; 5 Polish Society for Mountain Medicine and Rescue, Szczyrk, Poland; 6 Department of Disaster Medicine and Emergency Care, Jagiellonian University Medical College, Krakow, Poland; 7 Department of Combat Medicine, Military Institute, Warsaw, Poland; 8 Department of Emergency Medical Services, Medical University of Warsaw, Warsaw, Poland; 9 Department of Cardiac, Vascular and Transplantation Surgery, John Paul II Hospital, Jagiellonian University Medical College, Cracow, Poland

**Keywords:** Accidental hypothermia, Pulmonary ventilation, Capnography

## Abstract

**Background:**

Severe accidental hypothermia can cause circulatory disturbances ranging from cardiac arrhythmias through circulatory shock to cardiac arrest. Severity of shock, pulmonary hypoperfusion and ventilation-perfusion mismatch are reflected by a discrepancy between measurements of CO_2_ levels in end-tidal air (EtCO_2_) and partial CO_2_ pressure in arterial blood (PaCO_2_). This disparity can pose a problem in the choice of an optimal ventilation strategy for accidental hypothermia victims, particularly in the prehospital period. We hypothesized that in severely hypothermic patients capnometry should not be used as a reliable guide to choose optimal ventilatory parameters.

**Methods:**

We undertook a pilot, observational case-series study, in which we included all consecutive patients admitted to the Severe Hypothermia Treatment Centre in Cracow, Poland for VA-ECMO in stage III hypothermia and with signs of circulatory shock. We performed serial measurements of arterial blood gases and EtCO_2_, core temperature, and calculated a PaCO_2_/EtCO_2_ quotient.

**Results:**

The study population consisted of 13 consecutive patients (ten males, three females, median 60 years old). The core temperature measured in esophagus was 20.7–29.0 °C, median 25.7 °C. In extreme cases we have observed a Pa-EtCO_2_ gradient of 35–36 mmHg. Median PaCO_2_/EtCO_2_ quotient was 2.15.

**Discussion and Conclusion:**

Severe hypothermia seems to present an example of extremely large Pa-EtCO_2_ gradient. EtCO_2_ monitoring does not seem to be a reliable guide to ventilation parameters in severe hypothermia.

## Background

While end-tidal carbon dioxide (EtCO_2_) monitoring is one of the objective standards set in the Intensive Care Society guidelines [1, 2] and is of particular use for verification of endotracheal tube placement [1], it does not seem to be a reliable guide to ventilation in profound shock states.

It was noted that abnormal EtCO_2_ measurements on initial emergency department presentation correlate with bad prognosis both in adults and children [1]. Since cerebral blood vessels are sensitive to changes in partial pressure of CO_2_ (PaCO_2_), and hypocapnia induced by hyperventilation can lead to vasoconstriction and as a consequence worsening of secondary brain injury, it is advocated that ventilation parameters should be aimed at achieving “normocapnia”.

Pulmonary hypoperfusion and pulmonary ventilation – perfusion mismatch seem to play an important role among many factors determining extremely large Pa-EtCO_2_ gradient observed in severe hypothermia victims. This discrepancy is further aggravated by a drop in blood temperature itself.

There is no published data on Pa-EtCO_2_ gradient and reliability of EtCO_2_ measurement in severe hypothermia. Based on our experience we hypothesize that in severely hypothermic patients capnometry should not be used as a reliable guide to choose optimal ventilatory parameters.

## Methods

We carried out a retrospective observational case-series study. All patients admitted to the Severe Hypothermia Treatment Centre (SHTC-Cracow, Poland) with stage III hypothermia, that still had a circulation and features of shock, were enrolled [3]. All data analyzed was collected on admission. The measurement of the central temperature (Tc) was taken in the lower third esophagus, using single-use Smiths Medical 12Fr probes, coupled with a SpaceLab cardiomonitor. The value of EtCO_2_ was estimated from the main stream with the use of a capnometer from the SpaceLab monitoring system.

Blood tests were assayed by routine automated laboratory techniques (Radiometer Copenhagen model ABL80). Blood gas analyses according to alpha-stat (blood gases measured at 37 °C ) were performed in the central hospital laboratory, certified with a program by RIQAS (Randox Quality Assessment Scheme, UK). Simple plotting of PaCO_2_ against EtCO_2_ was performed. The study was approved by the Local Ethical Committee of the John Paul II Hospital in Cracow.

## Results

The study population consisted of 13 patients (ten males, three females, median 60 age years). The core temperature measured in the oesophagus was 20.7–29 °C, median 25.7 °C. PaCO_2_ values varied between 17 to 53,1 mmHg (median 25.5 mmHg), and EtCO_2_ from 12 to 19 mmHg (median 17 mmHg).

**Fig. 1:**
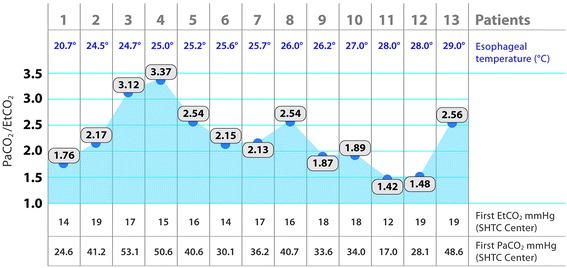
Pa-EtCO_2_ gradient parameters of patients in stage III hypothermia

In extreme cases we have observed a Pa-EtCO_2_ gradient of 35–36 mmHg. Median PaCO_2_/EtCO_2_ quotient was 2.15 (blood gases measured at 37 °C). Figure 1 summarizes the parameters of patients in stage III hypothermia who still had a circulation.

## Discussion

General guidelines for ventilatory support do not cover special population of severe hypothermia patients (Swiss Stage III and IV, Table 1) [4, 5]. Some experts recommend that the respiratory rate of mechanical ventilation should be lower [6], others prefer the ventilation rate to be normal [7]. The Wilderness Medical Society guidelines state that in intubated patients, without the possibility of EtCO_2_ control, it is recommended to decrease the respiratory rate by half in relation to the value in normothermia. At the same time, in patients in which capnometry is available, it is recommended to maintain EtCO_2_ in normal range [8]. In the latest review of the current knowledge about hypothermia, there is an emphasis on the maintenance of normocapnia in order to prevent arrhythmia related to hyper- or hypoventilation [9].

**Table 1:** Swiss Stage of Hypothermia

Hypothermia stage	Clinical findings	Core temperature (if available)
I (mild)	Conscious; shivering	35–32 °C
II (moderate)	Impaired consciousness; may or may not be shivering	<32–28 °C
III (severe)	Unconscious; vital signs present	<28 °C
IV	Vital signs absent	Variable

Maintenance of normoventillation and normocapnia in patients in hypothermia is not an easy task. In mild, therapeutic hypothermia, such as in the ICU, normocapnia is achieved and maintained in only about 55% [10]. Unfortunately, even the EtCO_2_ does not solve the problem. It has been ascertained that in mild, therapeutic hypothermia (36 – 32 °C), the gradient between PaCO_2_ and EtCO_2_ may increase 2,5-fold and be as high as 18.7 mmHg [11].

During the prehospital period, the only practical way to assess PaCO_2_ is by indirect measurement of end-tidal CO_2_ (EtCO_2_). In normotermia, the Pa- EtCO_2_ gradient is usually 4–6 mmHg, so the EtCO_2_ values may be easily treated as a baseline for establishing parameters of normoventilation.

However, in our opinion, in significantly decreased core temperatures this is an unreliable guide to ventilation because of the profound metabolic, circulatory and respiratory disturbances, especially within the ventilation - perfusion mismatch, which accompanies severe hypothermia. This has been confirmed by the results obtained in our patients.

The impact of hypothermia on the partial pressure of CO_2_ in arterial blood and acid-base balance has been known for years, especially in cardiac surgery. Two methods of interpreting blood gas results have been proposed. One is the pH-stat strategy in which ventilation is adjusted to maintain PaCO_2_ at 40 mmHg at the patient’s current body temperature. Such a correction is difficult to calculate, and is hardly ever used in the adult population [12]. Most centres, including ours, do not use this method, and prefer to use the alpha-stat strategy instead. In this approach, ventilation is adjusted to maintain PaCO_2_ at 40 mmHg at 37 °C, meaning that PaCO_2_ will be <40 mm Hg in hypothermia. The alpha-stat strategy is recommended nowadays for patients with hypothermia [5, 9, 13].

The difference between EtCO_2_ and PaCO_2_ (blood gases measured at 37 °C) are generally consistent with the observations conducted by Sitzwohl et al., although this study only looked at patients with mild intra-operative hypothermia (32–36 °C) [11]. The small number of observations obtained so far does not allow us to carry out statistical analysis. Nevertheless, there is a definite trend of increasing Pa-EtCO_2_ gradient above 1 as the core temperature falls, although the correlation is not linear, as presented on the Fig. 1. In our opinion, such a large gradient is a result of both increased CO_2_ solubility as temperature decreases and the increase in ventilation-perfusion disorders, including low cardiac output. Interestingly, Sitzwohl et al. found that the mode of ventilation did not have a significant effect on the Pa-EtCO_2_ gradient. Unfortunately, no data concerning the actual parameters of ventilation in the pre-hospital phase has been recorded in our patients, though we hope to obtain this data in a future study.

At present, it is unclear whether the recommendation to mildly hypoventilate patients with hypothermia has clinical significance [8]. It is worth noting that based on local guidelines, SHTC coordinators routinely recommend the use of normoventilation during transport as part of a lung-protective ventilation strategy (Vt = 6–7 ml/kg of ideal body weight, PEEP 5 mmHg and ventilatory rate = 10/min), and it should be regarded as the optimal ventilation strategy in adults. We also advocate for avoidance of manual bag-valve ventilation due to its tendency to hyperventilate.

EtCO_2_ monitoring in hypothermic victims should be used not only as proof of correct positioning of the endotracheal tube, but also, as a sign of preserved pulmonary flow, while circulatory instability is reflected by a fall of EtCO_2_. Critically low values suggest cardiac arrest. Such observation may be particularly valuable in the event of cardiac arrest with pulseless electrical activity (PEA), whose confirmation can be very difficult in the pre-hospital phase, where no ultrasound and invasive blood pressure measurement are available.

## Conclusions

A very high Pa-EtCO_2_ gradient is found in patients with severe hypothermia.

In patients with severe hypothermia, the EtCO_2_ values should not be used as the main criterion for the selection of ventilatory parameters.

The optimal ventilatory technique in patients with hypothermia should be mechanical lung protective ventilation.
